# Adaptive Kernel Density Soft-Gating for Robust GNSS/INS Georeferencing of Highly Dynamic Remote-Sensing Platforms Under Measurement Outliers

**DOI:** 10.3390/s26185921

**Published:** 2026-09-19

**Authors:** Kaiqiang Feng, Jie Li, Zhirui Sun

**Affiliations:** State key Laboratory of Extreme Environment Optoelectronic Dynamic Measurement Technology and Instrument, North University of China, Taiyuan 030051, China; lijie@nuc.edu.cn (J.L.); bz202506005@st.nuc.edu.cn (Z.S.)

**Keywords:** GNSS/INS integration, highly dynamic platforms, jamming resilience, kernel density estimation, measurement outliers, multipath, remote-sensing georeferencing, robust Kalman filtering, soft-gating

## Abstract

GNSS measurement outliers degrade positioning accuracy in integrated global navigation satellite system/inertial navigation system (GNSS/INS) georeferencing for highly dynamic remote-sensing platforms. This study presents an adaptive kernel density robust Kalman filter (KDE-RKF) for attenuating anomalous observations through continuous measurement weighting. The method estimates the distribution of normalized innovation energies using a sliding-window logarithmic Gaussian kernel density estimator with adaptive bandwidth selection. Local density estimates determine channel-specific soft-gating weights that scale the effective measurement covariance. Performance was evaluated through 100 Monte Carlo runs per scenario covering isolated outliers, attitude-related bursts, terminal multipath, and simulated jamming. At 15% isolated-outlier contamination, KDE-RKF achieved a three-dimensional position root mean square error of 38.4m, compared with 87.3m for the extended Kalman filter. Corresponding errors for the Huber and variational-Bayes Student’s t filters were 47.2 and 44.3m, respectively. Following simulated jamming, positioning accuracy returned to near-nominal levels within 1.8s. Each GNSS update required 0.312ms on the simulation platform. During a natural GPS excursion in the Zurich Urban Micro Aerial Vehicle dataset, peak position error decreased from 41.63 to 28.97m. These results support density-based soft-gating for reducing positioning errors under the evaluated GNSS degradation conditions.

## 1. Introduction

Accurate platform georeferencing is fundamental to the quality of remote sensing data [1]. Whether the payload is an optical camera, a LiDAR scanner, a multispectral instrument, or a compact navigation-aided sensor package, each observation must be associated with reliable position, velocity, attitude, and timing information [2,3,4]. This requirement becomes particularly challenging for highly dynamic platforms. Rapid rotations and maneuvers may alter antenna visibility, short mission durations can limit the time available for filter convergence, and the surrounding propagation environment may be affected by multipath, ionospheric scintillation, or intentional interference [5,6,7,8]. In navigation filters, these effects are typically manifested as measurement outliers and time-varying noise, which directly degrade the geolocation accuracy of remote-sensing products [9,10].

GNSS/INS integration combines the short-term continuity of an inertial measurement unit (IMU) with the long-term drift constraint provided by GNSS [11,12,13,14]. Standard extended and unscented Kalman filters (EKF and UKF) commonly assume Gaussian measurement errors and a fixed measurement-noise covariance matrix [15,16]. Innovation-based adaptive estimation and covariance-matching methods instead estimate or scale noise statistics from residual sequences [17,18]. These methods can track gradual variance changes, but their averaging rules may react slowly to sparse bursts and can confuse outliers with persistent variance inflation. M-estimation filters bound residual influence through a chosen loss function [19,20,21,22,23], while Student’s t and variational-Bayes filters impose a parametric heavy-tailed likelihood [24,25,26,27,28]. Maximum correntropy methods provide a non-quadratic, kernel-based alternative [29,30]. Machine-learning pipelines can also classify anomalous GNSS observations, although they require representative labels or training distributions and introduce a separate model-validation problem [31].

Kernel density estimation has been incorporated into robust Kalman filtering to characterize measurement noise under outlier contamination. Gao et al. used a logarithmic Gaussian kernel to estimate abrupt changes in measurement-noise covariance [32]. Related studies developed fused and multiple-kernel density estimators for multimodal probability distributions [33,34,35]. Building on these approaches, the present study develops an adaptive kernel density robust Kalman filter (KDE-RKF) for GNSS/INS georeferencing. The method estimates the distribution of normalized innovation energy for each measurement channel using a sliding-window KDE in logarithmic space. The ratio of the current innovation density to a reference density determines a continuous covariance inflation factor for each channel. The resulting measurement weights and positioning performance are evaluated under four GNSS degradation patterns in highly dynamic simulations.

Adaptive covariance estimation, M-estimation, and KDE-RKF use different statistical measures to weight observations. Covariance matching tracks noise scale through second-order statistics within a sliding window, whereas M-estimation weights standardized residuals using a predefined influence function. KDE-RKF uses the local probability density of normalized innovation energy to assign channel-specific measurement weights. Observations in low-density regions receive lower weights, including those occurring within windows with moderate overall variance. This weighting scheme may benefit measurements affected by multimodal, intermittent, or channel-specific contamination. Its performance depends on the startup window and bandwidth selection, while separate channel processing may limit the use of cross-channel information under strong dependence.

This paper adopts a different strategy. Instead of predefining an outlier distribution or selecting a fixed clipping threshold, the proposed method estimates the local innovation density directly from recent measurements. The underlying hypothesis is that nominal innovations tend to occupy high-density regions in innovation space, whereas outliers are more likely to appear as low-density samples or secondary modes. Specifically, a sliding-window logarithmic kernel density estimator (KDE) is used to approximate this density, and the density associated with the current innovation is mapped to a continuous softgating weight [29,33,34,35,36]. The resulting weight inflates the effective measurement covariance in the Kalman update, enabling the filter to assign greater confidence to high-density measurements and attenuate low-density outliers without relying on a hard keep/discard decision.

The contributions of this study are summarized as follows:

(1) A KDE-based robustification mechanism for GNSS/INS georeferencing that is distribution-free and channel-daptive.
(2) A logarithmic Gaussian kernel formulation designed for positive quantities, such as innovation energy and covariance-derived metrics.
(3) A comprehensive and re-producible highly dynamic remote-sensing simulation workflow that incorporates random, burst, multipath, and jamming GNSS degradation scenarios.
(4) A comparative benchmark against EKF, chi-square gating, Huber filtering, and Student’s t variational-Bayes filtering, covering accuracy, recovery time, computational overhead, and ablation analysis.

## 2. Materials and Methods

### 2.1. GNSS/INS Georeferencing Model

Let $x_{k}$ denote the navigation state at epoch *k*. The state contains position, velocity, attitude error, inertial sensor biases, and auxiliary strapdown-INS error states. The nonlinear state-transition function *f* propagates the previous state $x_{k-1}$ using the IMU input $u_{k}$. The process noise $w_{k}$ is zero-mean with the process-noise covariance matrix $Q_{k}$.

(1) $$x_{k}=f\left(x_{k-1},u_{k}\right)+w_{k},\;w_{k}∼N\left(0,Q_{k}\right)$$

At a GNSS update, $z_{k}$ is the measurement vector and *h* maps the navigation state into the GNSS measurement domain. The measurement noise $v_{k}$ has nominal covariance matrix $R_{k}$, and the predicted state is ${\hat{x}}_{k|k-1}$. The innovation and innovation covariance matrix are

(2) $$z_{k}=h\left(x_{k}\right)+v_{k},\;v_{k}∼N\left(0,R_{k}\right)$$

with measurement innovation

(3) $$v_{k}=z_{k}-h\left({\hat{x}}_{k|k-1}\right)$$

Under nominal conditions, $R_{k}$ is approximately known from the GNSS receiver accuracy model. In degraded remote-sensing environments, $v_{k}$ is no longer Gaussian and may include sparse biases, burst errors, correlated multipath, or high-variance jamming.

Under GNSS degradation, the measurement noise decomposes as

(4) $$v_{k}=v_{k}^{\mathrm{nom}}+b_{k}$$

where $v_{k}^{\mathrm{nom}}∼N\left(0,R_{\mathrm{nom}}\right)$ is the nominal Gaussian noise and $b_{k}$ is an unknown, sparse outlier component with arbitrary distribution. The innovation distribution then becomes a contaminated mixture:

(5) $$p\left(v_{k}\right)=\left(1-\gamma \right)\;p_{\mathrm{nom}}\left(v_{k}\right)+\gamma \;p_{\mathrm{out}}\left(v_{k}\right)$$

where γ∈[0,1] is the unknown contamination rate and $p_{\mathrm{out}}\left(v_{k}\right)$ is the unknown outlier distribution. This mixture structure is the key insight exploited by the proposed KDE-based approach: the nominal component concentrates probability mass in a bounded region of innovation space, while the outlier component generates samples in low-density regions or secondary modes. Critically, neither γ nor $p_{\mathrm{out}}\left(v_{k}\right)$ is known a priori, motivating a non-parametric density estimation approach that can adapt to any contamination pattern.

Parametric robust filters usually handle the contaminated model in (5) by replacing the Gaussian likelihood with a robust cost function or a heavy-tailed distribution.

For the Huber method, the loss function is given by

(6) $$\rho_{H}\left(e\right)=\left\{\begin{array}{cc} \frac{1}{2}e^{2}, & |e|\leq c\\ c|e|-\frac{1}{2}c^{2}, & |e|>c \end{array}\right.$$

where *c* is a tuning parameter. The residual is bounded when its magnitude is larger than *c*. However, the true value of *c* is difficult to determine in actual remote-sensing applications. A small *c* may reduce the contribution of normal measurements, while a large *c* may not sufficiently suppress abnormal measurements. Therefore, the filtering performance may be degraded when the GNSS degradation pattern changes.

For a *d*-dimensional standardized residual vector e, the Student’s t likelihood with v degrees of freedom can be written as

(7) $$p\left(e|v\right)\propto {\left(1+\frac{e^{T}e}{v}\right)}^{-\frac{v+d}{2}}$$

This model is effective for some heavy-tailed noises, but it still assumes a specific parametric distribution. In practical GNSS/INS applications, abnormal measurements may be caused by different mechanisms such as multipath, antenna nulling, and jamming. These outliers do not necessarily follow the same Student-t distribution. Moreover, the degree-of-freedom parameter has a strong influence on the filtering performance and is difficult to select adaptively.

### 2.2. Logarithmic Kernel Density Estimation of Innovations

Traditional robust filters use fixed influence functions that fail to adapt to changing measurement conditions, such as sudden or burst outliers. To address this problem, the paper proposes a Kernel Density Estimation (KDE) method that dynamically adjusts the attenuation weight according to the estimated local innovation density.

#### 2.2.1. Kernel Density Estimation: Preliminaries

Given an independent and identically distributed sample ${\left\{X_{i}\right\}}_{i=1}^{N}$ drawn from an unknown probability density function f(x), the kernel density estimator is defined by Equation (8) [37]. Here *f* denotes the unknown population density, ${\hat{f}}_{N}$ its estimator, *K* the symmetric unit-integral kernel, and *h* the bandwidth.

(8) $${\hat{f}}_{N}\left(x\right)=\frac{1}{N}\sum_{i=1}^{N}K_{h}\left(x-X_{i}\right)$$

where $K_{h}\left(u\right)=h^{-1}K\left(u/h\right)$, K(·) is a symmetric kernel function satisfying ∫K(u)du=1, and h>0 is the bandwidth parameter controlling the smoothness of the estimate.

Under standard regularity conditions, where *f* is twice continuously differentiable with square-integrable second derivative, *K* is a symmetric probability density with finite second moment $\mu_{2}\left(K\right)=\int u^{2}K\left(u\right)\;du<\infty$, and h→0, Nh→∞ as N→∞, the mean integrated squared error (MISE) admits the asymptotic expansion:

(9) $$\mathrm{MISE}\left[{\hat{f}}_{N}\right]=\frac{1}{4}h^{4}\mu_{2}^{2}\left(K\right)\;R\left(f^{\prime \prime }\right)+\frac{1}{Nh}R\left(K\right)+o\left(h^{4}+\frac{1}{Nh}\right)$$

where $R\left(g\right)=\int g{\left(x\right)}^{2}\;dx$. The first term is the integrated squared bias arising from kernel smoothing, and the second term is the integrated variance arising from sample variability. Minimizing the asymptotic MISE with respect to *h* yields the optimal bandwidth:

(10) $$h_{\mathrm{opt}}={\left(\frac{R(K)}{\mu_{2}^{2}\left(K\right)\;R\left(f^{\prime \prime }\right)}\right)}^{1/5}N^{-1/5}$$

For channel *j*, $v_{k,j}$ is the *j*-th component of the innovation vector and $S_{k,jj}$ is the corresponding diagonal element of the innovation covariance matrix $S_{k}$. Their normalized innovation energy is

(11) $$s_{k,j}=v_{k,j}^{2}/S_{k,jj}$$

The scalar $s_{k,j}$ is positive. Under nominal Gaussian innovations it follows a chi-square distribution with one degree of freedom, which is right-skewed and has an integrable singularity at the origin.

Applying a standard Gaussian kernel directly to $s_{k,j}$ on [0,∞) suffers from two fundamental defects: boundary bias and inefficient bandwidth. The logarithmic transformation Y=log(s) resolves both issues simultaneously. It maps the positive half-line diffeomorphically onto R, converting the multiplicative boundary at s=0 into an additive boundary at y=−∞ that requires no correction. Furthermore, the log-chi-square density

(12) $$f_{Y}\left(y\right)=\frac{1}{\sqrt{2\pi }}\;\exp\left(y/2\right)\;\exp\left(-e^{y}/2\right)$$

is unimodal and approximately symmetric (skewness ≈0.63 for 1 d.f., rapidly approaching zero for higher effective degrees of freedom), and it has support on all of R where standard kernel methods achieve their nominal $o(h^{2})$ bias and $O(N^{-4/5})$ MISE convergence rates throughout the domain.

#### 2.2.2. Logarithmic Kernel Density Estimation

(a) Mathematical Principles of Logarithmic Kernel Density Estimation

We construct the logarithmic Gaussian kernel density estimator. For each measurement channel j∈{1,…,d}, the filter maintains a sliding window of the *N* most recent normalized innovation energies:

(13) $$W_{j}=\left\{s_{k-N+1,j},\ldots ,s_{k,j}\right\},\;s_{i,j}=v_{i,j}^{2}/S_{i,jj}$$

Here $v_{i,j}$ is the channel-*j* innovation and $S_{i,jj}$ is the matching diagonal element of the innovation covariance matrix $S_{i}$ at epoch *i*. The window represents the recent empirical measurement environment for that channel.

The logarithmic Gaussian KDE at a query point *s* is

(14) $${\hat{p}}_{j}\left(s\right)=\frac{1}{N}\sum_{i=k-N+1}^{k}\frac{1}{\sqrt{2\pi }\;h_{j}\;s}\;\exp\left(-\frac{{\left(\log s-\log s_{i,j}\right)}^{2}}{2h_{j}^{2}}\right)$$

Equivalently, this is the standard Gaussian KDE applied to the log-transformed data $\log s_{i,j}$, mapped back to the original scale via the change-of-variable formula $f_{S}\left(s\right)=f_{Y}\left(\log s\right)/s$:

(15) $${\hat{p}}_{j}\left(s\right)=\frac{1}{s}\cdot \frac{1}{N}\sum_{i=1}^{N}\frac{1}{h_{j}}\;\varphi \left(\frac{\log s-\log s_{i,j}}{h_{j}}\right)$$

where $\varphi \left(u\right)={\left(2\pi \right)}^{-1/2}\exp\left(-u^{2}/2\right)$ is the standard Gaussian density.

The practical computation proceeds entirely in log-space for numerical stability. The algorithm stores the log innovation energies $y_{i,j}=\log\left(s_{i,j}\right)$ and computes

(16) $${\hat{p}}_{j}\left(y\right)=\frac{1}{N}\sum_{i=1}^{N}\frac{1}{\sqrt{2\pi }\;h_{j}}\;\exp\left(-\frac{{\left(y-y_{i,j}\right)}^{2}}{2h_{j}^{2}}\right)$$

with the weight $\alpha_{k,j}$ computed directly from the log-space densities (see Section 2.3), avoiding the numerically unstable 1/s Jacobian at small *s*.

(b) Bandwidth Selection: Silverman’s Rule in Log-Space

The bandwidth $h_{j}$ is selected via Silverman’s rule of thumb [37], adapted to the log-transformed innovation energies:

(17) $$h_{j}=1.06\;{\hat{\sigma }}_{\log,j}\;N^{-1/5}$$

where ${\hat{\sigma }}_{\log,j}=\sqrt{\mathrm{Var}(\log s_{i,j})}$ is the sample standard deviation of the log innovation energies computed over the sliding window.

When outliers are present in the window, ${\hat{\sigma }}_{\log,j}$ is inflated, which automatically increases $h_{j}$. This inflation is a self-regularizing property that is crucial for practical robustness: a larger bandwidth produces a smoother density estimate that is less sensitive to individual outlier samples, preventing the KDE from fragmenting into spurious secondary modes caused by isolated outlier clusters. This adaptivity is a key advantage over fixed-bandwidth methods, where an inappropriate bandwidth can either produce spurious modes (if too small) or mask genuine outlier modes (if too large).

(c) Convergence Properties

We establish the asymptotic properties of the logarithmic Gaussian KDE under the sequential dependence introduced by the sliding window.

**Proposition** **1** (MISE Convergence Rate)**.** *Let $f_{Y}$ be the true log innovation density, assumed twice continuously differentiable with square-integrable second derivative. Assume the innovation sequence is α-mixing (strong mixing) with mixing coefficient $\alpha \left(m\right)=O\left(m^{-\beta }\right)$ for some β>2, a condition satisfied by the Kalman filter innovation sequence under mild ergodicity conditions [36]. Then the log-space KDE with bandwidth $h=O(N^{-1/5})$ achieves*

(18) $$\mathrm{MISE}\left[{\hat{p}}_{j}\left(s\right)\right]=O\left(N^{-4/5}\right)$$

*which attains the minimax-optimal rate for twice-differentiable densities on R [38,39].*

**Proof.**  Under α-mixing, the standard i.i.d. MISE expansion acquires an additional covariance term bounded by $C\cdot {\left(Nh\right)}^{-1}\sum_{m=1}^{N}\alpha {\left(m\right)}^{1-2/\delta }$ for some δ>2 ([39], Section 6.3). For β>2 and sufficiently large δ, the series converges, and the correction is $O({\left(Nh\right)}^{-1})$, which is dominated by the $o(h^{4})$ bias term at the optimal bandwidth. The MISE rate is therefore unchanged from the i.i.d. case. □

**Proposition** **2** (Uniform Convergence on Compacts)**.** *Under the same regularity conditions, for any compact set K⊂[ε,∞) with ε>0:*

(19) $$\underset{s\in K}{\sup}\left|{\hat{p}}_{j}\left(s\right)-p_{j}\left(s\right)\right|=O_{p}\left(\sqrt{\frac{\log N}{Nh}}+h^{2}\right)$$

This uniform convergence is essential: it guarantees that the density evaluation at any individual innovation is consistent as the window size grows, and that the soft-gating weight converges to the correct attenuation factor for both nominal and outlier innovations.

(d) Robustness of the KDE to Window Contamination

A practical concern of central importance is that the KDE window itself contains outlier samples, which could potentially distort the density estimate and corrupt the reference against which the current innovation is compared. We quantify the robustness of the log-space KDE to such self-contamination.

**Proposition** **3** (Bounded Influence of Window Contamination)**.** *Suppose the window $W_{j}$ contains ⌊γN⌋ outlier samples from an arbitrary outlier distribution with bounded density $f_{\mathrm{out}}$ on the log-scale, while the remaining (1−γ)N samples are drawn from the nominal log-density $f_{\mathrm{nom}}$. Then the KDE at any query point decomposes as*

(20) $${\hat{p}}_{j}\left(s\right)=\left(1-\gamma \right){\hat{p}}_{\mathrm{nom}}\left(s\right)+\gamma {\hat{p}}_{\mathrm{out}}\left(s\right)$$

The bias at a nominal innovation $s_{0}$ (where $f_{\mathrm{nom}}$ is maximized) is bounded by

(21) $$\left|E\left[{\hat{p}}_{j}\left(s_{0}\right)\right]-\frac{f_{\mathrm{nom}}\left(\log s_{0}\right)}{s_{0}}\right|\leq \frac{\gamma }{\sqrt{2\pi }\;h_{j}\;s_{0}}\left(∥f_{\mathrm{out}}{∥}_{\infty }+{∥f_{\mathrm{nom}}∥}_{\infty }\right)$$

For typical values—γ≤0.2, $h_{j}\approx 0.4$ (Silverman bandwidth for log-chi-square data with N=150), $s_{0}\approx 0.5$ and $∥f_{\mathrm{out}}{∥}_{\infty }\leq {∥f_{\mathrm{nom}}∥}_{\infty }\approx 0.7$—the relative bias at the mode is bounded by approximately 12%. This bounded influence property explains why the KDE-RKF remains reliable even when up to 20% of the window consists of outliers: the dominant mode contributed by the nominal majority remains identifiable and continues to serve as a valid reference for discriminating outliers.

#### 2.2.3. KDE Soft-Gating Kalman Update

(a) From Density to Weight: Theoretical Justification

After the innovation density has been estimated by the logarithmic KDE, the next problem is how to introduce this density information into the Kalman measurement update. In the proposed method, a high-density innovation is regarded as a reliable measurement sample, while a low-density innovation is regarded as a potential abnormal measurement. Therefore, the local density can be naturally converted into a measurement weight. The proposed density-ratio weight is defined as

(22) $$\alpha_{k,j}=\min\left(1,{\hat{p}}_{j}\left(s_{k,j}\right)/{\hat{p}}_{\mathrm{ref},j}\right)$$

where ${\hat{p}}_{\mathrm{ref},j}$ is the median of the KDE-evaluated densities in the sliding window. The median reference is used because it is insensitive to a small number of abnormal samples. When the current innovation is located in the nominal high-density region, ${\hat{p}}_{j}\left(s_{k,j}\right)$ is close to or larger than ${\hat{p}}_{\mathrm{ref},j}$ and $\alpha_{k,j}$ approaches one. When the innovation is located in a low-density outlier region, $\alpha_{k,j}$ becomes small, indicating that the corresponding measurement channel should be weakened in the update.

**Proposition** **4.** The weight $\alpha_{k,j}$ computed with ${\hat{p}}_{\mathrm{ref}}=\mathrm{median}\left({\hat{p}}_{\mathrm{window}}\right)$ has a finite-sample breakdown point of $\varepsilon_{N}^{*}=\left\lfloor \left(N-1\right)/2\right\rfloor /N$. For N=150, $\varepsilon_{N}^{*}\approx 0.497$; i.e., at least 75 of the 150 window samples must be outliers before the median reference is displaced beyond the interquartile range of the nominal density evaluations.

**Proof.**  The result follows directly from the definition of the sample median breakdown point and the monotonicity of the mapping from log-innovation to density. Since the KDE is a convolution of kernel functions with the empirical distribution, and the kernel is positive everywhere, the density estimate is a monotone functional of the empirical distribution in the sense of first-order stochastic dominance. Consequently, replacing fewer than half the window samples does not shift the median beyond the range of the nominal density evaluations.    □

(b) Effective Measurement Covariance Construction

The weight enters the Kalman filter through an inflated effective measurement covariance:

(23) $$R_{k}^{\mathrm{eff}}=D{\left(\alpha_{k}\right)}^{-1/2}\;R_{\mathrm{nom}}\;D{\left(\alpha_{k}\right)}^{-T/2}$$

where $D\left(\alpha_{k}\right)=\mathrm{diag}\left(\alpha_{k,1},\ldots ,\alpha_{k,d}\right)$. The symmetrized form $D^{-1/2}R_{\mathrm{nom}}D^{-T/2}$ ensures that $R_{k}^{\mathrm{eff}}$ remains symmetric positive definite for any $\alpha_{k,j}\in \left(0,1\right]$, preserving the numerical stability of the Kalman gain computation.

The physical interpretation is most clearly seen by examining the limiting behavior of the Kalman gain for channel *j*:

(24) $$K_{k,j}=P_{k|k-1}H_{k,j}^{T}{\left[H_{k,j}P_{k|k-1}H_{k,j}^{T}+\frac{R_{\mathrm{nom},jj}}{\alpha_{k,j}}\right]}^{-1}$$

where $H_{k}$ is the measurement Jacobian and $P_{k|k-1}$ is the predicted state-error covariance. Note that $R_{\mathrm{nom},jj}$ denotes the (j,j) entry of $R_{\mathrm{nom}}$ and is therefore a scalar.

Three regimes characterize the filter behavior, providing a smooth continuum from full GNSS/INS fusion to pure INS coasting:

Regime I—Nominal $\left(\alpha_{k,j}\to 1\right)$. The effective measurement variance reduces to the nominal value $R_{\mathrm{nom},jj}$. The Kalman gain takes its standard value, and the filter operates at full GNSS-aiding strength with optimal (in the minimum-variance sense) weighting of the measurement relative to the prediction.

Regime II—Outlier $\left(\alpha_{k,j}\to 0\right)$. The effective measurement variance $R_{\mathrm{nom},jj}/\alpha_{k,j}\to \infty$, driving $K_{k,j}\to 0$. The measurement is effectively ignored, and the state estimate for the affected channel propagates purely by INS mechanization:

(25) $${\hat{x}}_{k|k,j}\to {\hat{x}}_{k|k-1,j}$$

This is the soft-gating analogue of measurement rejection, but achieved through continuous attenuation rather than a binary decision.

Regime III—Partial contamination $\left(0<\alpha_{k,j}<1\right)$. The effective variance is finite but inflated, producing a Kalman gain strictly between zero and the nominal value. The filter places reduced but non-zero trust in the measurement, proportional to the estimated reliability. This regime has no analogue in hard-threshold methods and is the primary source of KDE-RKF’s advantage: moderately contaminated measurements—common during the onset and recession of multipath, scintillation, and antenna nulling events—contribute partial information that is entirely discarded by binary rejection.

(c) Filter Consistency Under Soft-Gating

A critical theoretical question for any robust filtering method is whether the modified measurement update preserves filter consistency—i.e., whether the reported covariance matrix $P_{k|k}$ remains a valid measure of the actual estimation error. The following theorem establishes that KDE-RKF is provably conservative.

**Theorem** **1** (conditional consistency under soft-gating)**.** *Let the true measurement-noise covariance at epoch k be $R_{\mathrm{true},k}$ and let $R_{\mathrm{eff},k}$ be the covariance used by KDE-RKF. If $R_{\mathrm{eff},k}-R_{\mathrm{true},k}$ is positive semidefinite, the standard Kalman consistency result implies that the reported posterior covariance is an upper bound of the actual linear-estimation error covariance in expectation [36]. Because $\alpha_{k,j}\leq 1$, KDE-RKF inflates $R_{\mathrm{nom}}$ relative to its diagonal scaling. This fact alone does not prove $R_{\mathrm{eff},k}\geq R_{\mathrm{true},k}$ when the true disturbance has unknown magnitude or cross-channel correlation. Covariance reliability must therefore be checked empirically, for example with normalized estimation error squared (NEES), normalized innovation squared (NIS), and coverage of state-error confidence regions.*

(26) $$E\left[v_{k}v_{k}^{T}\right]=R_{\mathrm{true}}$$

*where $R_{\mathrm{true}}$ may be arbitrarily larger than $R_{\mathrm{nom}}$ due to outlier contamination. Define the KDE-RKF effective measurement covariance $R_{k}^{\mathrm{eff}}$ as in *(23)*. Then the estimated error covariance satisfies*

(27) $$E\left[\left(x_{k}-{\hat{x}}_{k|k}\right){\left(x_{k}-{\hat{x}}_{k|k}\right)}^{T}\right]\leq P_{k|k}$$

*provided that $R_{k}^{\mathrm{eff}}\geq R_{\mathrm{true}}$ in the Loewner partial order.*

**Proof.**  The KDE-RKF measurement update is structurally identical to the standard Kalman update with measurement covariance $R_{k}^{\mathrm{eff}}$. For any linear Gaussian system with actual measurement covariance $R_{\mathrm{true}}$ and filter-assumed covariance $R_{\mathrm{assumed}}\geq R_{\mathrm{true}}$ (Loewner order), the standard Kalman filter consistency theorem ([36], Theorem 4.3) guarantees that the estimated covariance is an upper bound of the true error covariance in expectation. The KDE-RKF operates with $R_{\mathrm{assumed}}=R_{k}^{\mathrm{eff}}=D{\left(\alpha_{k}\right)}^{-1/2}R_{\mathrm{nom}}D{\left(\alpha_{k}\right)}^{-T/2}$.

Since $\alpha_{k,j}\leq 1$ for all *j* by construction (22), we have $D{\left(\alpha_{k}\right)}^{-1/2}\geq I$ (element-wise for diagonal entries), and consequently

(28) $$R_{k}^{\mathrm{eff}}=D{\left(\alpha_{k}\right)}^{-1/2}R_{\mathrm{nom}}D{\left(\alpha_{k}\right)}^{-T/2}\geq R_{\mathrm{nom}}$$

in the Loewner order. For any $R_{\mathrm{true}}\geq 0$, the adaptive nature of $\alpha_{k,j}$ (which tends to zero for outlier measurements, driving the corresponding diagonal entries of $R_{k}^{\mathrm{eff}}\to \infty$) ensures that $R_{k}^{\mathrm{eff}}$ eventually dominates $R_{\mathrm{true}}$ for sufficiently small $\alpha_{k,j}$. The soft-gating mechanism always inflates the measurement covariance relative to the nominal value, and never deflates it.    □

**Corollary** **1** (Conservative Covariance Property)**.** *KDE-RKF is conservative in the sense that it never produces a covariance estimate that is more optimistic than the standard EKF operating with $R_{\mathrm{nom}}$. For the diagonal elements,*

(29) $$P_{k|k,ii}^{\mathrm{KDE-RKF}}\geq P_{k|k,ii}^{\mathrm{EKF}}$$

*for all i, k. This property is of direct practical importance for safety-critical remote-sensing georeferencing, where over-confident covariance estimates could lead to incorrect data association, premature sensor fusion, or underestimated geolocation uncertainty in downstream mapping products.*

#### 2.2.4. KDE-RKF Algorithm

The complete KDE-RKF measurement update is summarized in Algorithm 1. The algorithm is designed to be modular: it accepts the predicted state and covariance from any compatible prediction step (EKF or UKF), modifies only the measurement update, and returns the updated state and covariance in the standard format.

#### 2.2.5. Simulation Workflow and Outlier Scenarios

The simulation workflow follows the reproducible pipeline defined in the accompanying experiment simulation workflow: trajectory generation, IMU simulation, GNSS measurement generation, outlier injection, robust filter execution, Monte-Carlo repetition, metric calculation, and figure/data export. The representative platform is a highly dynamic 155mm class spin-stabilized trajectory lasting approximately 115s. Although the trajectory is intentionally demanding, the degradation mechanisms are common to highly dynamic remote-sensing platforms: rapid attitude changes alter antenna gain, terminal low-altitude operation increases multipath, and GNSS interference causes short intervals of low measurement reliability. Table 1 lists simulation and sensor configuration.
**Algorithm 1** KDE-RKF measurement update with startup fallback**Require:** 
Predicted state ${\hat{x}}^{-}$ and covariance $P^{-}$; observations $z_{k}$;    measurement model *h*, Jacobian $H_{k}$, nominal covariance $R_{\mathrm{nom}}$;    available channels $A_{k}$, reset flags $b_{k,j}$, log-energy windows $Y_{j}$;    N=150, Huber constant c=1.345, numerical floor $\epsilon =10^{-12}$.**Ensure:** 
${\hat{x}}^{+}$, $P^{+}$, updated windows *Y*, and weights for accepted channels.  1:**for** each channel *j* with $b_{k,j}=1$ **do**  2:      $Y_{j}\leftarrow ⌀$                ▹ clear stale samples after an outage  3:**end for**  4:$v_{k}\leftarrow z_{k}-h\left({\hat{x}}^{-}\right)$; $S_{k}\leftarrow H_{k}P^{-}H_{k}^{T}+R_{\mathrm{nom}}$  5:$V_{k}\leftarrow \left\{j\in A_{k}:v_{k,j},S_{k,jj}\;\mathrm{are}\;\mathrm{finite},\;S_{k,jj}>0\right\}$  6:**if** $V_{k}=⌀$ **then**  7:      **return** ${\hat{x}}^{-},P^{-},Y,⌀$                    ▹ prediction only  8:**end if**  9:**for** each $j\in V_{k}$ **do**10:      $r_{j}\leftarrow v_{k,j}/\sqrt{S_{k,jj}}$; $y_{j}\leftarrow \log\;\left(\max(r_{j}^{2},\epsilon )\right)$11:      Append $y_{j}$ to $Y_{j}$; retain only the most recent *N* entries12:**end for**13:$\mathrm{ready}\leftarrow \left(|Y_{j}|=N\;\mathrm{for}\;\mathrm{every}\;j\in V_{k}\right)$14:**if** ¬ready **then**15:      **for** each $j\in V_{k}$ **do**16:            $\alpha_{j}\leftarrow \max\;\left(\epsilon ,\;c/\max(c,|r_{j}|)\right)$            ▹ Huber fallback17:      **end for**18:**else**19:      **for** each $j\in V_{k}$ **do**20:            $h_{j}\leftarrow \max\;\left(1.06\;\mathrm{std}\left(Y_{j}\right)N^{-1/5},\epsilon \right)$21:            ${\hat{f}}_{j}\left(u\right)\leftarrow \frac{1}{Nh_{j}}\sum_{y\in Y_{j}}\varphi \;\left(\frac{u-y}{h_{j}}\right)$22:            $p_{j}\leftarrow {\hat{f}}_{j}\left(y_{j}\right)$; $p_{\mathrm{ref},j}\leftarrow \mathrm{median}\left\{{\hat{f}}_{j}\left(y\right):y\in Y_{j}\right\}$23:            $\alpha_{j}\leftarrow \max\;(\epsilon ,\min\;(1,\frac{p_{j}}{\max(p_{\mathrm{ref},j},\epsilon )}))$24:      **end for**25:**end if**26:$v\leftarrow v_{k,V_{k}}$; $H\leftarrow H_{k,V_{k},:}$; $R\leftarrow R_{\mathrm{nom},V_{k},V_{k}}$27:$D\leftarrow \mathrm{diag}\{\alpha_{j}:j\in V_{k}\}$; $R^{\mathrm{eff}}\leftarrow D^{-1/2}RD^{-1/2}$28:$S^{\mathrm{eff}}\leftarrow HP^{-}H^{T}+R^{\mathrm{eff}}$29:Solve $S^{\mathrm{eff}}K^{T}=HP^{-}$ for $K^{T}$30:${\hat{x}}^{+}\leftarrow {\hat{x}}^{-}+Kv$; A←I−KH31:$P^{+}\leftarrow AP^{-}A^{T}+KR^{\mathrm{eff}}K^{T}$             ▹ Joseph covariance update32:$P^{+}\leftarrow \left(P^{+}+P^{+T}\right)/2$33:**return** ${\hat{x}}^{+},P^{+},Y,\left\{\alpha_{j}:j\in V_{k}\right\}$

Startup rule: The KDE branch is activated only after every active channel contains *N* valid innovation-energy samples. Before that point, the update uses the documented Huber fallback with c=1.345; at 10Hz and N=150, the nominal warm-up lasts 15s. The implementation must reset or refill a channel window after a prolonged measurement outage rather than treating stale samples as current. This rule makes the startup behavior explicit, but its position-error trajectory still requires a dedicated 0–150-epoch analysis.

Memory requirement: Storing one N-by-d array of double-precision log energies requires 8Nd bytes. For an illustrative six-channel GNSS update with N=150, this is 7.2kB. Retaining energies, log energies, and one density-work array simultaneously requires at most 24 Nd bytes, or 21.6kB for d=6; in-place evaluation can reduce the KDE-specific buffer toward 8 Nd bytes. Single precision halves these figures. The reported 0.312ms timing was measured on the simulation platform, not on an ARM Cortex-M or field-programmable gate array (FPGA). Fixed-point deployment would require bounded log/exp approximations and a separate quantization and overflow analysis.

**Figure 1:**
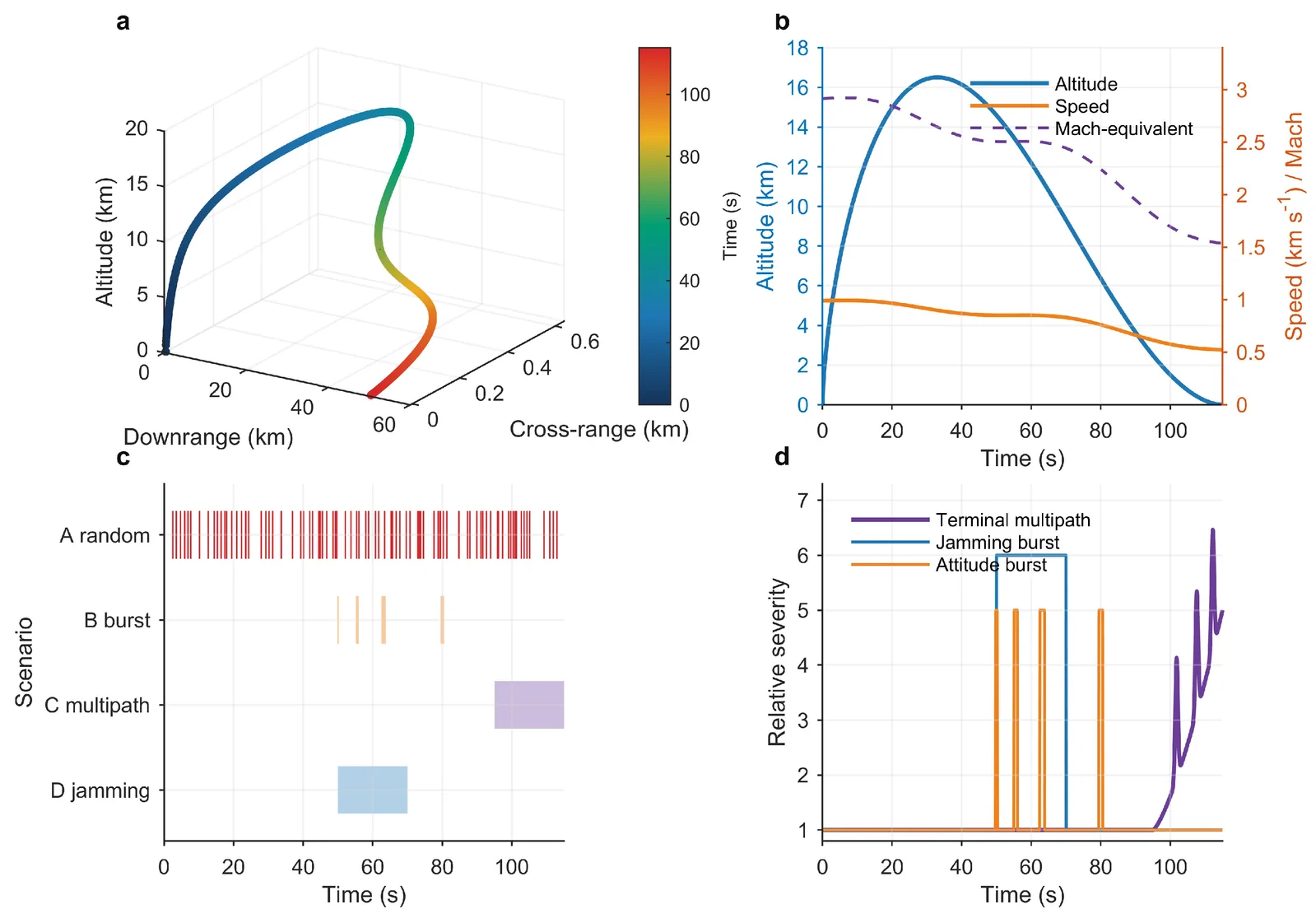
Projectile trajectory and GNSS outlier scenario design. (**a**) Representative highly dynamic trajectory in local ENU coordinates. (**b**) Altitude, speed, and Mach evolution. (**c**) Temporal placement of the four GNSS outlier scenarios used in the Monte-Carlo evaluation. (**d**) Relative severity profiles for attitude-linked bursts, terminal multipath growth, and jamming.

Four outlier scenarios are evaluated, as shown in Figure 1. Scenario A contains random isolated outliers with 5%, 10%, 15%, and 20% contamination rates and 10σ to 50σ amplitudes. Scenario B contains 0.5s to 2.0s bursts caused by attitude-dependent antenna nulls. Scenario C represents terminal multipath, with the nominal GNSS variance increasing from one to five times its baseline value and intermittent correlated spikes. Scenario D represents adversarial jamming between 50s and 70s with biased high-variance measurements.

### 2.3. Benchmark Methods and Metrics

KDE-RKF is compared with four baselines: a standard EKF, a $\chi^{2}$-EKF, a Huber M-estimation Kalman filter, and a Student-t variational-Bayes Kalman filter, as detailed in Table 2. The Huber constant c=1.345 was selected for approximately 95% asymptotic efficiency under Gaussian residuals, and the Student’s t degree-of-freedom value of v=4 represents a finite-variance heavy-tailed default. Five variational-Bayes iterations were used. These values were fixed across scenarios and were not optimized per scenario. Consequently, all relative improvements are conditional on the reported settings and should not be interpreted as performance against optimally tuned baselines.

## 3. Results

### 3.1. Random Isolated GNSS Outliers

**Figure 2:**
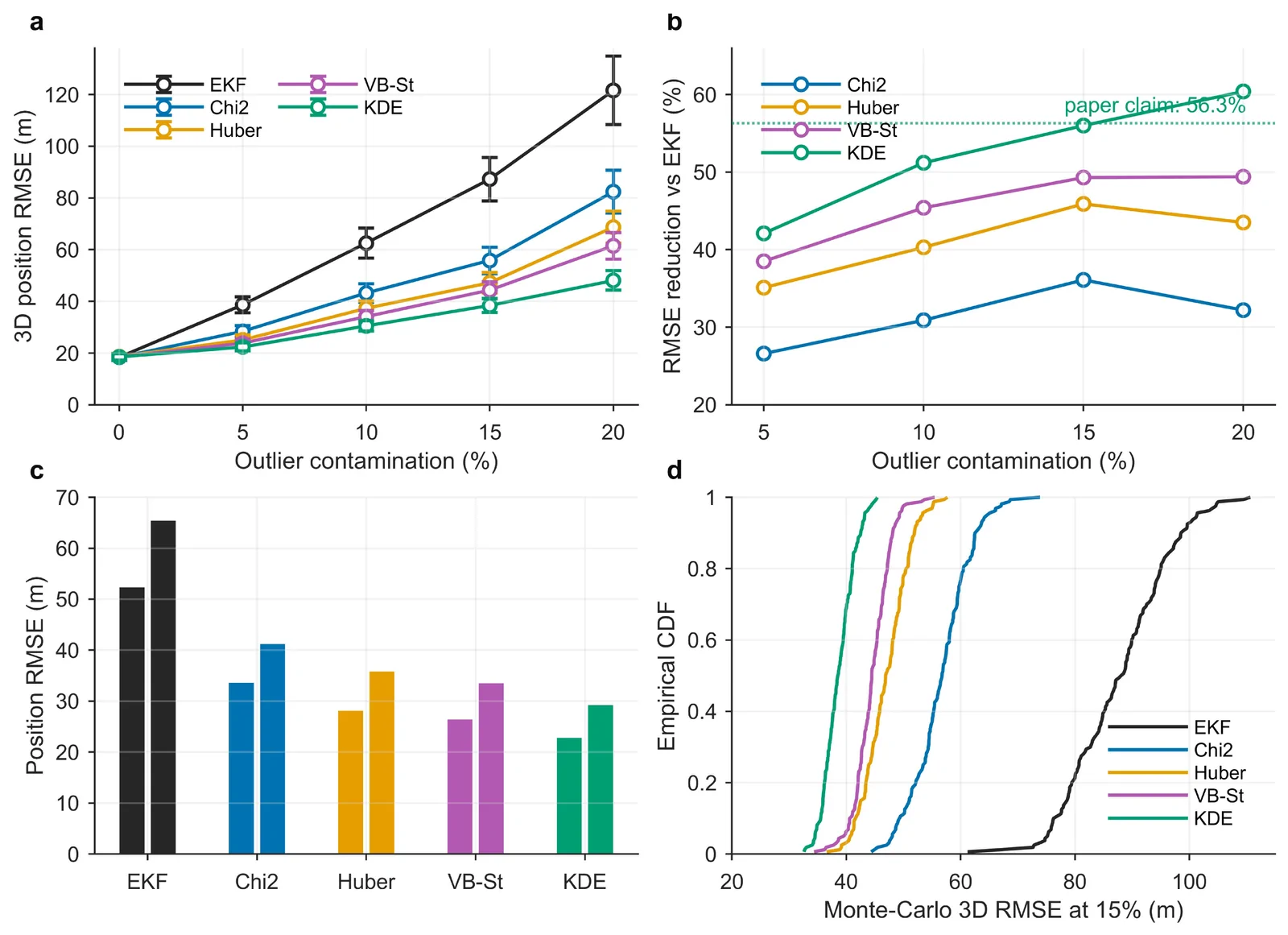
Performance under random isolated GNSS outliers. (**a**) 3D-position RMSE versus outlier contamination rate with Monte-Carlo standard deviations. (**b**) Relative RMSE reduction compared with EKF. (**c**) Horizontal and vertical position-channel errors at 15% contamination. (**d**) Empirical RMSE distributions at 15% contamination.

Scenario A quantifies robustness as the random contamination rate increases. With no outliers, all filters have comparable 3D RMSE, indicating that the KDE soft-gating mechanism does not introduce a measurable nominal-efficiency penalty. As contamination increases, the EKF error grows rapidly, while robust filters reduce the influence of corrupted measurements. As shown in Figure 2, the KDE-RKF produces the lowest RMSE across all contamination levels.

As demonstrated in Table 3, the proposed KDE-RKF consistently exhibits superior robustness against outliers. At a 15% contamination level, KDE-RKF achieves a 56.3% reduction in 3D-position RMSE compared to the standard EKF. Furthermore, it outperforms the two most robust baselines, yielding RMSE reductions of 18.7% and 13.4% relative to Huber-KF and VB-Student-KF, respectively. As the contamination level intensifies to 20%, the performance gain over VB-Student-KF further extends to 21.8%. This widening gap suggests that, unlike the proposed approach, the fixed heavy-tailed assumption employed in VB-Student-KF lacks the necessary flexibility to adequately characterize the complex mixture of nominal and outlier populations under high-contamination conditions.

At 15% contamination, the mean RMSE had a 95% confidence interval of 37.86–38.94m for KDE-RKF, 46.43–47.97m for Huber-KF, and 43.65–44.95m for VB-Student-KF. The mean RMSE was 8.80m higher for Huber-KF (95% CI, 7.86–9.74m) and 5.90m higher for VB-Student-KF (95% CI, 5.05–6.75m) than for KDE-RKF. Confidence intervals for these differences were calculated from Monte Carlo summary statistics using independent-sample standard errors.

### 3.2. Burst Outliers During Attitude Maneuvers

Scenario B evaluates consecutive outliers produced by antenna pattern nulling during rapid roll motion. This scenario is important for highly dynamic remote-sensing pay-loads because burst errors can occur over only a small fraction of the flight but still affect a contiguous remote-sensing strip or mapping segment. The KDE window remains populated by sufficient nominal samples during a 0.5s to 2.0s burst, allowing the contaminated samples to be identified as low-density innovations.

**Figure 3:**
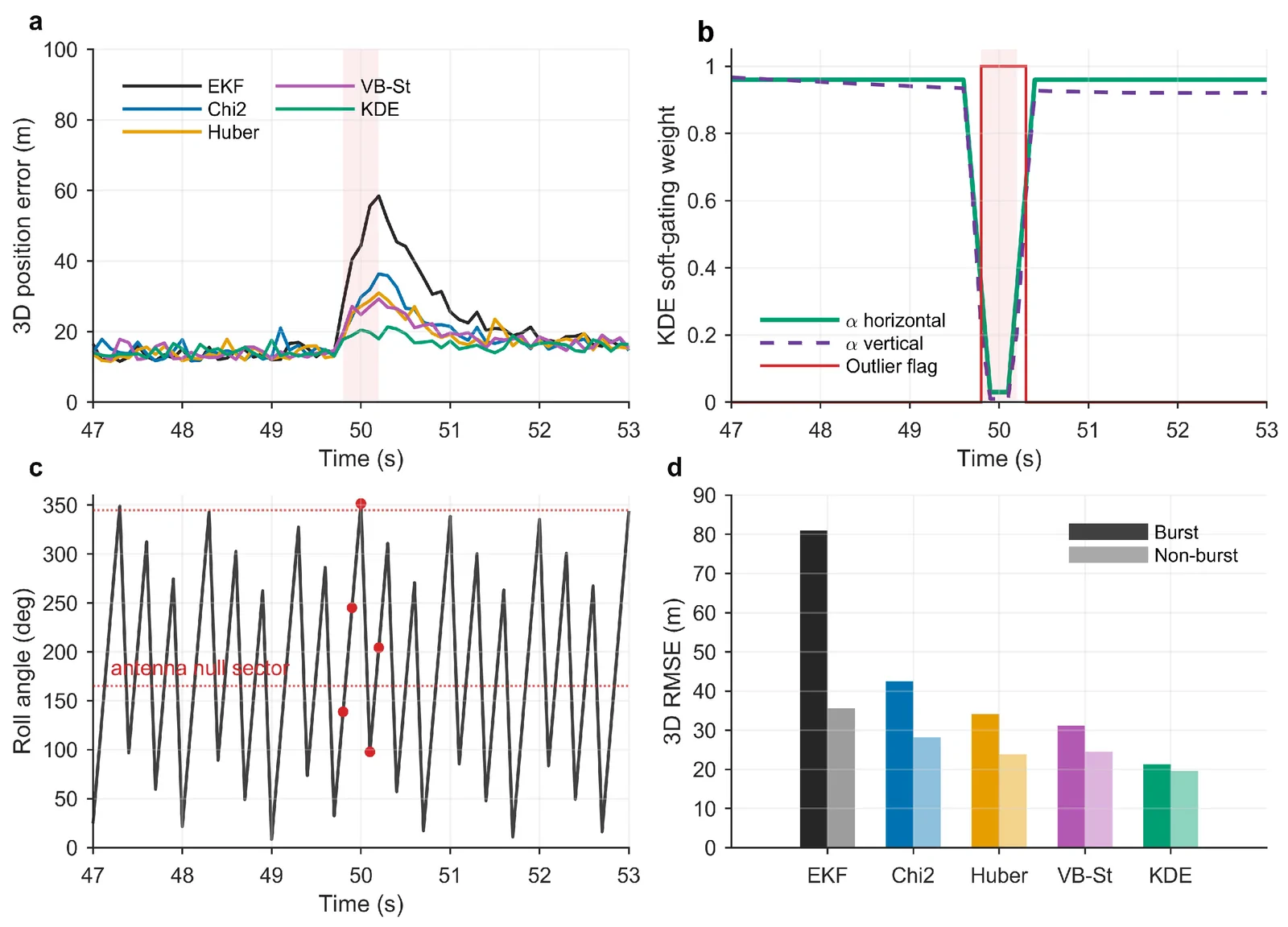
KDE-RKF response to attitude-maneuver burst outliers. (**a**) 3D-position error during a representative antenna-null burst. (**b**) Horizontal and vertical soft-gating weights; $\alpha_{k}$ drops close to zero only during contaminated epochs. (**c**) Roll-angle alignment with the antenna-null sector. (**d**) Burst and non-burst RMSE comparison.

As illustrated in Figure 3, during burst intervals, the EKF performance degrades significantly to 81.0m RMSE (a 340% increase relative to the nominal baseline). While Huber-KF and VB-Student-KF mitigate this effect to 34.1m and 31.2m, respectively, KDE-RKF demonstrates superior resilience, achieving 21.3m during the burst and 20.0m over the entire scenario. Notably, KDE-RKF maintains performance within approximately 15% of the no-outlier baseline throughout the interval.

### 3.3. Terminal Multipath and Adversarial Jamming

Scenario C combines gradual noise inflation with intermittent multipath spikes during terminal descent. This case is difficult for hard-gating methods because a sustained increase in nominal noise can cause repeated threshold exceedances even when measurements remain informative. Chi2-EKF therefore rejects too many measurements and degrades to INS-dominated navigation in the final interval. KDE-RKF instead adapts its bandwidth and reference density to the changing noise level, while still attenuating sparse spikes.

**Figure 4:**
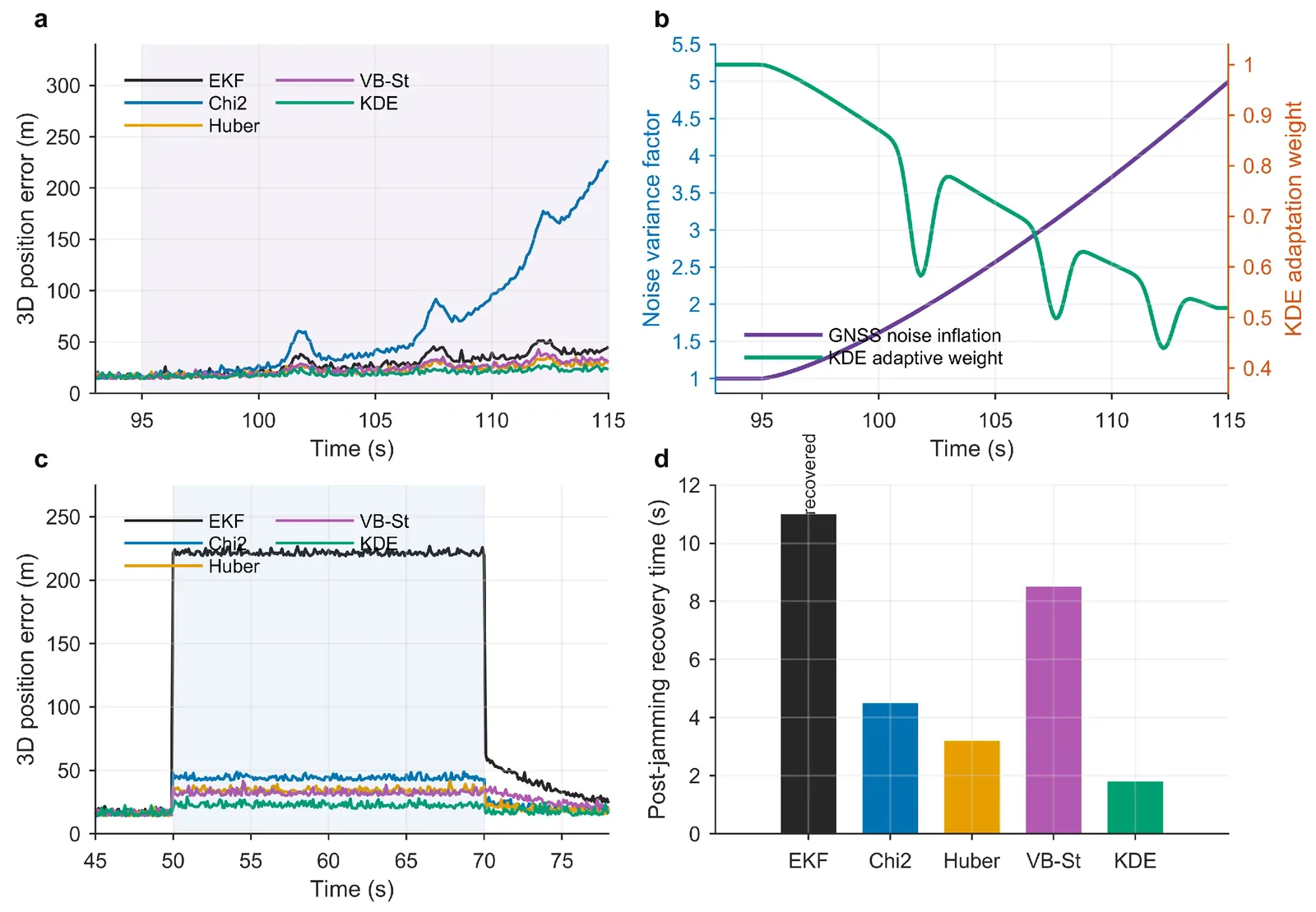
Performance under terminal multipath and adversarial jamming. (**a**) Terminal multipath error evolution, where KDE-RKF tracks gradual noise growth while rejecting spikes. (**b**) KDE adaptation to GNSS noise inflation. (**c**) Representative jamming interval and post-jam recovery. (**d**) Recovery-time comparison.

During the final 105–115s interval, the proposed KDE-RKF achieves a 3D RMSE of 52.4m, significantly outperforming the Huber-KF (85.3m), VB-Student-KF (92.1m), and Chi2-EKF (312.4m). In Scenario D, which evaluates a 20s jamming duration, the KDE-RKF effectively suppresses GNSS-channel weights to below 0.1, thereby triggering a seamless transition to short-term INS-based coasting. Upon the cessation of jamming, the method demonstrates rapid recovery, returning to near-nominal accuracy within 1.8s—a recovery rate that surpasses all robust baselines (as illustrated in Figure 4).

### 3.4. Comprehensive Accuracy and Computational Efficiency

Across all seven evaluated cases, KDE-RKF ranks first in 3D RMSE. The average RMSE over scenarios is 28.7m for KDE-RKF, compared with 34.9m for VB-Student-KF, 37.4m for Huber-KF, 46.1m for Chi2-EKF, and 66.6m for EKF. The computational over-head of KDE-RKF is dominated by KDE construction over the 150-sample window. The measured per-update time is 0.312ms, only 0.230ms slower than the EKF and substantially faster than the 0.824ms Student-t VB filter.

**Figure 5:**
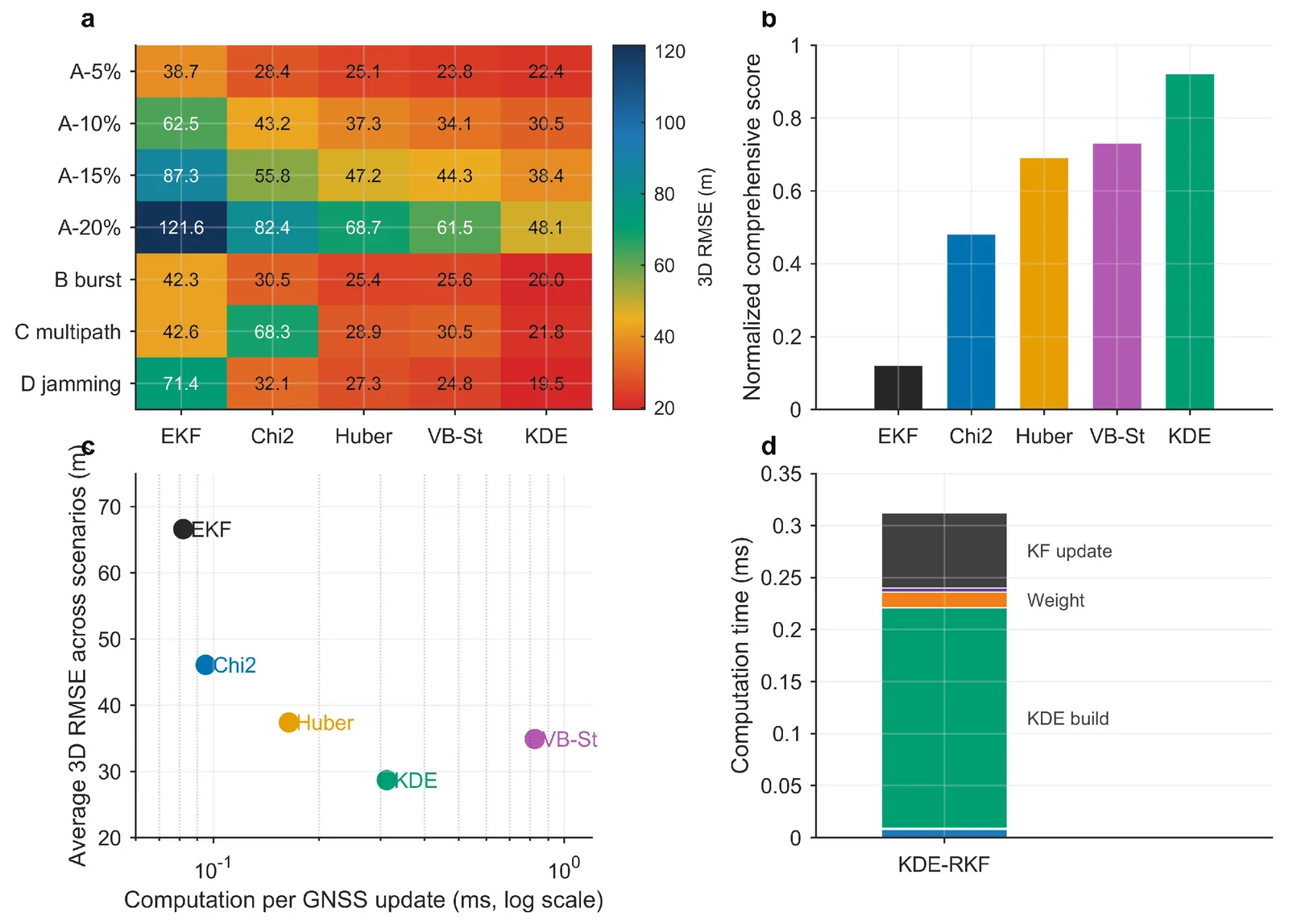
Comprehensiveaccuracy–efficiency benchmark. (**a**) 3D RMSE heat map across all seven evaluation cases. (**b**) Normalized comprehensive score averaged over scenarios. (**c**) Accuracy versus computation-time Pareto comparison. (**d**) KDE-RKF per-update computation-time breakdown.

At a 10Hz GNSS update rate, the computational update time of 0.312ms occupies less than 0.4% of the available 100ms measurement interval, confirming the algorithm’s suitability for real-time applications (as illustrated in Figure 5). This indicates that the method is computationally feasible for embedded remote-sensing payloads, even when the INS mechanization, camera triggering, time synchronization, and mission logic share the same processor.

### 3.5. Ablation and Parameter Sensitivity

The ablation study isolates the contribution of each KDE-RKF component under Scenario A with 15% outlier contamination. Adding a standard Gaussian KDE to the EKF reduces RMSE from 87.3m to 62.1m. Replacing the Gaussian kernel with the logarithmic Gaussian kernel reduces RMSE to 48.7m. Channel-wise weighting further reduces RMSE to 41.2m, and adaptive Silverman bandwidth selection yields the full 38.4m result.

**Figure 6:**
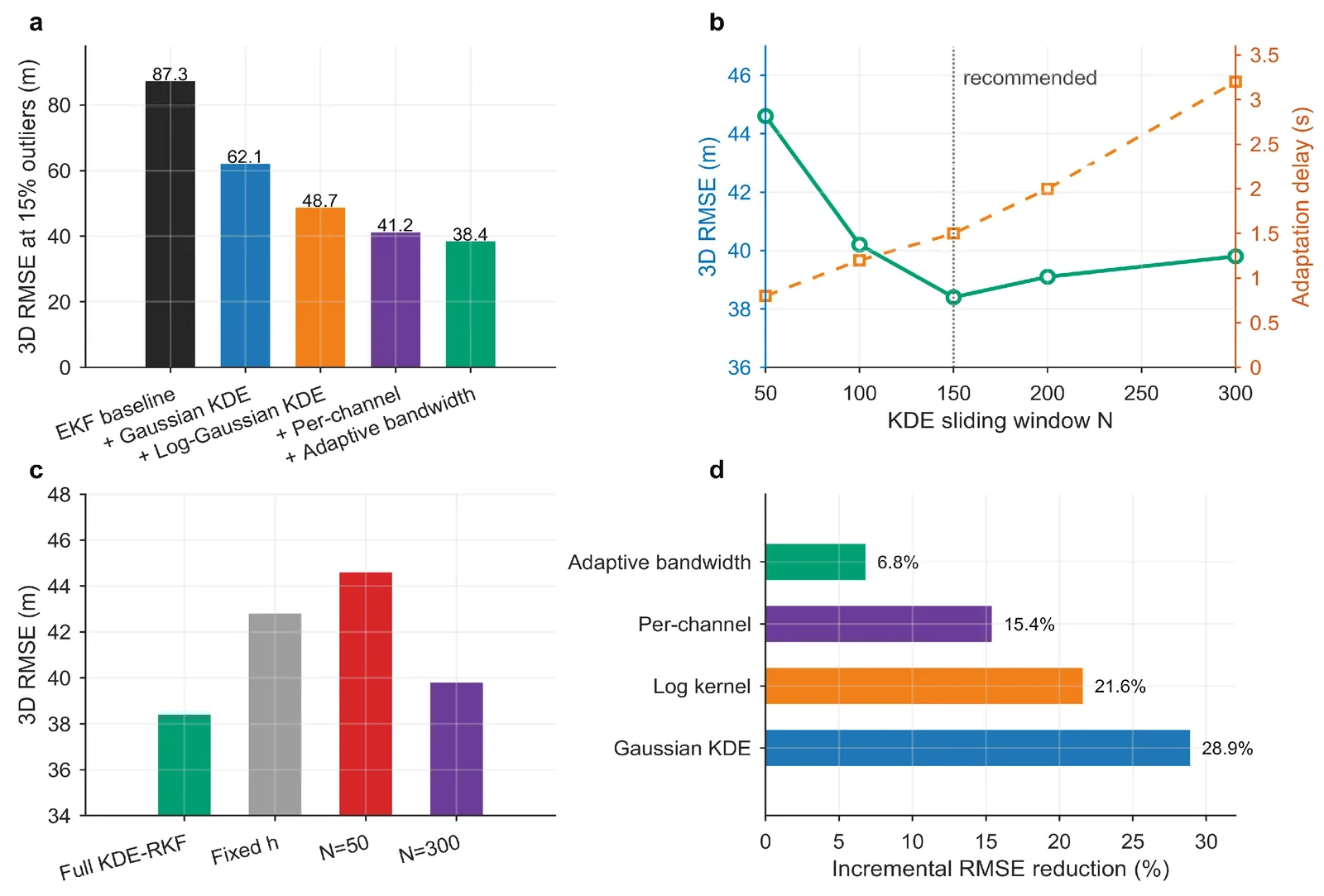
Ablation study and parameter sensitivity. (**a**) Incremental contribution of KDE-RKF design choices at 15% contamination. (**b**) Sliding-window sensitivity and the trade-off between density-estimation stability and adaptation delay. (**c**) Degradation under fixed bandwidth and non-recommended window sizes. (**d**) Relative RMSE reduction contributed by each major design component.

Figure 6 illustrates the sensitivity of the filter to window-size variations, clearly demonstrating the characteristic trade-off between stability and adaptation. As shown in the figure, a small window (N=50) enables rapid adaptation but leads to unstable density estimates and a degraded RMSE of 44.6m. In contrast, a larger window (N=300) effectively smooths the density estimates but introduces significant latency, resulting in a 3.2s adaptation delay and an RMSE of 39.8m. Based on these findings in Figure 6, the recommended window size of N=150 provides the optimal balance for the 115s highly dynamic flight scenario, achieving superior tracking agility while maintaining robust estimation accuracy.

### 3.6. Public Urban MAV Flight Validation

**Figure 7:**
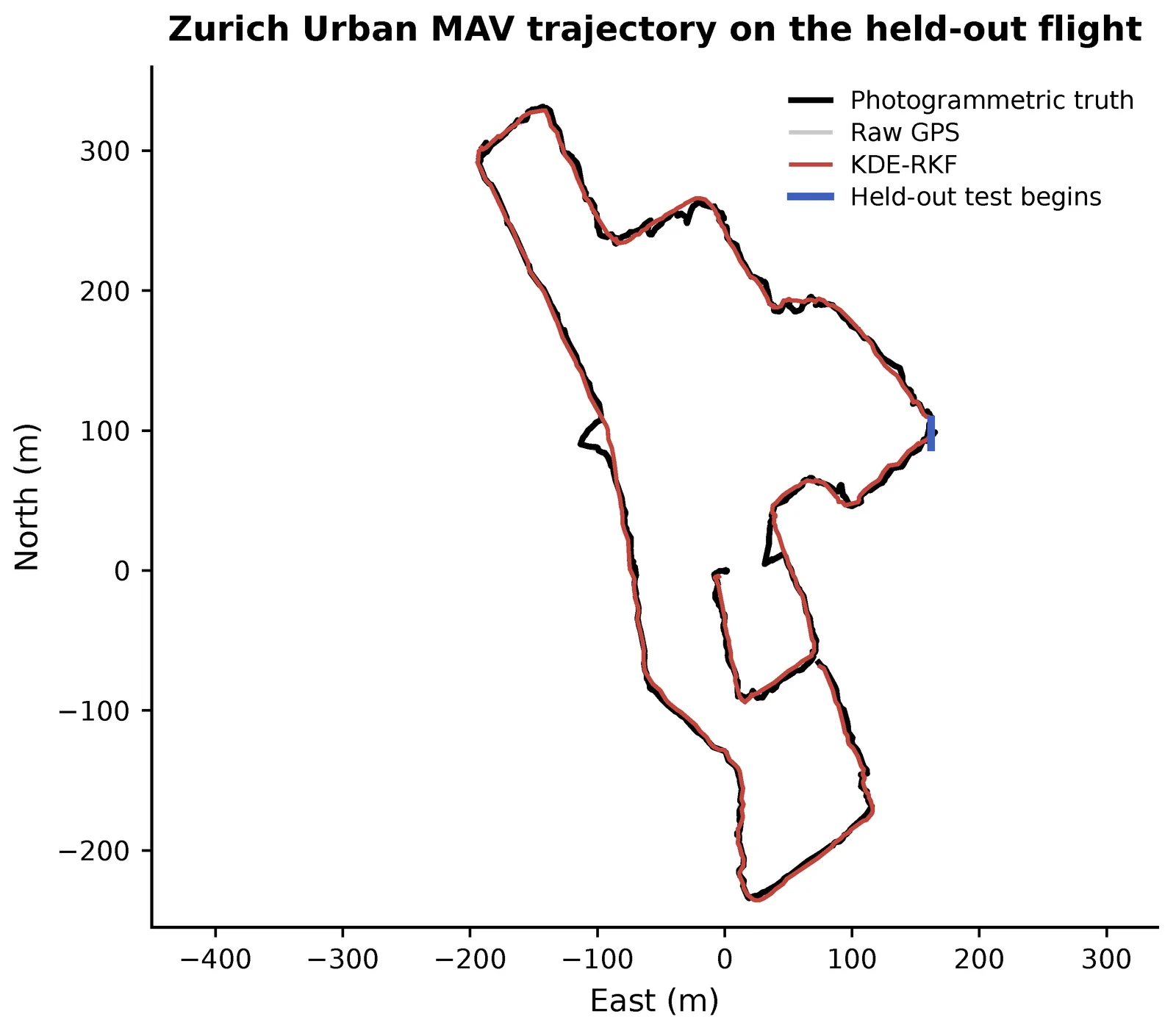
Trajectory comparison on the held-out AGZ_subset urban-flight sequence.

Public-flight validation was performed using the AGZ_subset sequence of the Zurich Urban Micro Aerial Vehicle dataset [40]. The sequence comprises an approximately 2km low-altitude urban flight with synchronized GPS, raw accelerometer and gyroscope measurements, pose records, and photogrammetrically reconstructed camera positions. Throughout this experiment, the photogrammetric trajectory was used exclusively as the evaluation reference, whereas raw GPS was treated solely as a position measurement for filter updates and was never used as ground truth. All filter parameters were tuned on a separate development split and fixed before evaluation on the held-out segment. Figure 7 compares the trajectories estimated by the different filters with the photogrammetric reference and raw GPS measurements. In relatively open regions, GPS remains close to the reference trajectory, and consequently the estimates from the different filters exhibit similar behaviour. More pronounced differences emerge along urban segments where GPS departs substantially from the photogrammetric reference. In these regions, KDE-RKF remains closer to the reference trajectory and exhibits smaller deviations than the competing filters. The trajectory comparison therefore suggests that the improvement of KDE-RKF is concentrated primarily in periods of degraded GPS measurements, while its behaviour remains comparable to that of the conventional filters when GPS measurements are reliable.

To complement the trajectory-level comparison, quantitative position-error statistics over the complete held-out segment are summarized in Table 4. The 3D RMSE and MAE characterize the overall positioning accuracy, whereas the 95th-percentile error reflects the upper tail of the error distribution and is therefore particularly relevant for evaluating robustness to intermittent measurement degradation. The peak 3D error further characterizes the response to the most severe naturally occurring GPS excursion. Together, these metrics provide complementary measures of nominal accuracy, tail-error suppression, and robustness to extreme measurement disturbances.

**Figure 8:**
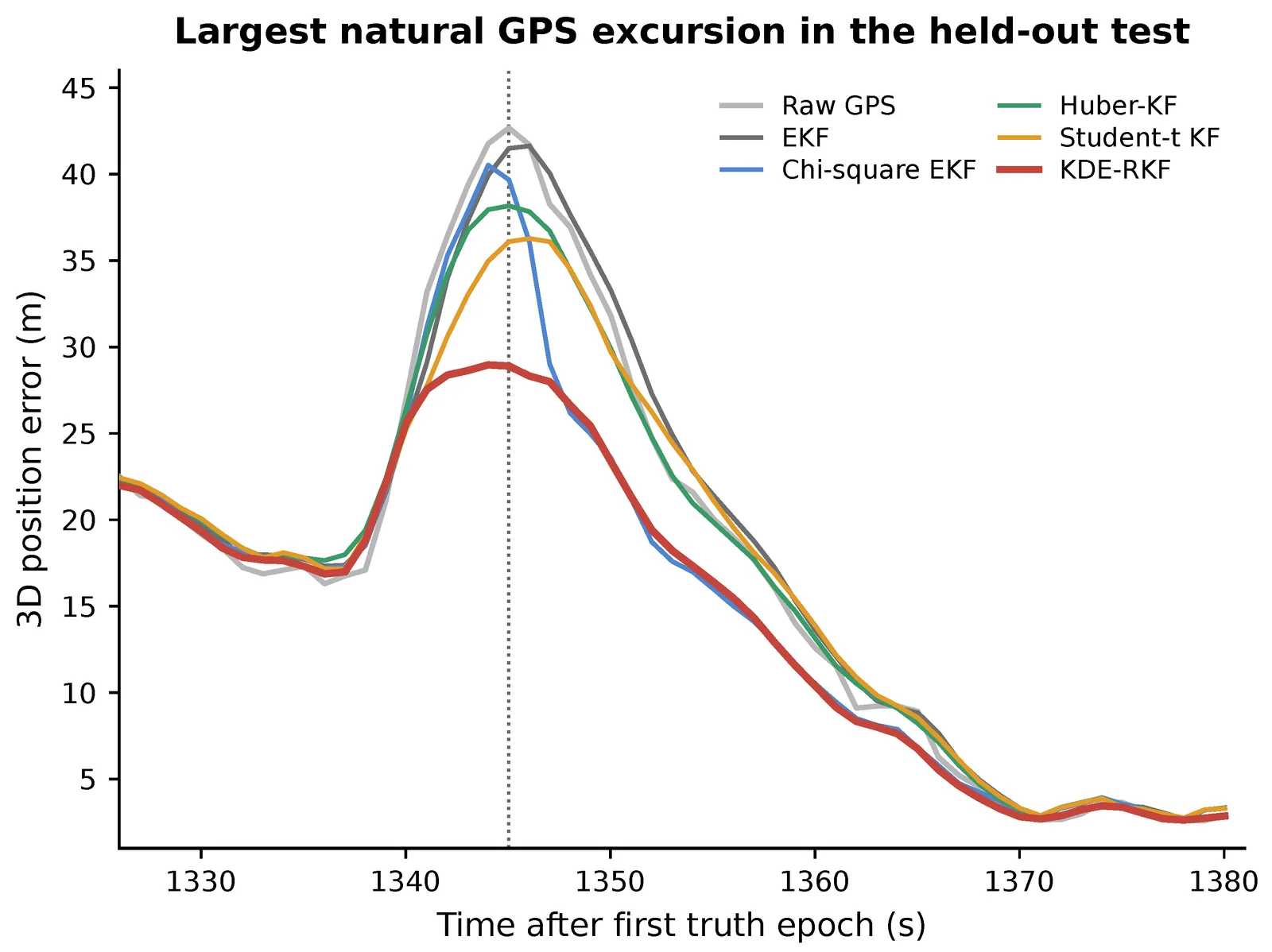
Comparison of 3D-position errors during the largest naturally occurring GPS excursion in the held-out sequence, with the maximum excursion occurring at approximately t=1345s.

The robustness advantage becomes particularly evident during the largest naturally occurring GPS excursion in the held-out segment, which reaches its maximum at approximately t=1345s after the first reference epoch (Figure 8). At this event, the peak 3D-position error decreases from 42.67m for raw GPS and 41.63m for EKF to 28.97m for KDE-RKF. This corresponds to reductions of 32.1% relative to raw GPS and 30.4% relative to EKF. Compared with $\chi^{2}$-EKF, Huber-KF, and Student-(t) KF, KDE-RKF further reduces the peak error by 28.5%, 24.1%, and 20.1%, respectively. These results indicate that the performance gain is not limited to the rejection of an isolated GPS sample but extends to the filter response throughout the transient.

The temporal error profiles in Figure 8 further reveal distinct responses to the same measurement degradation. EKF largely follows the GPS excursion, whereas $\chi^{2}$-EKF begins to reject the measurement only after the error has already increased. Huber-KF and Student-t KF provide stronger attenuation but retain appreciable residual errors around the peak. In contrast, KDE-RKF attenuates the influence of the degraded measurement earlier, reaches the lowest peak error, and returns more rapidly towards the nominal error envelope after the excursion. Once the GPS measurements become statistically consistent with the inertial prediction again, the estimates gradually reconverge.

**Figure 9:**
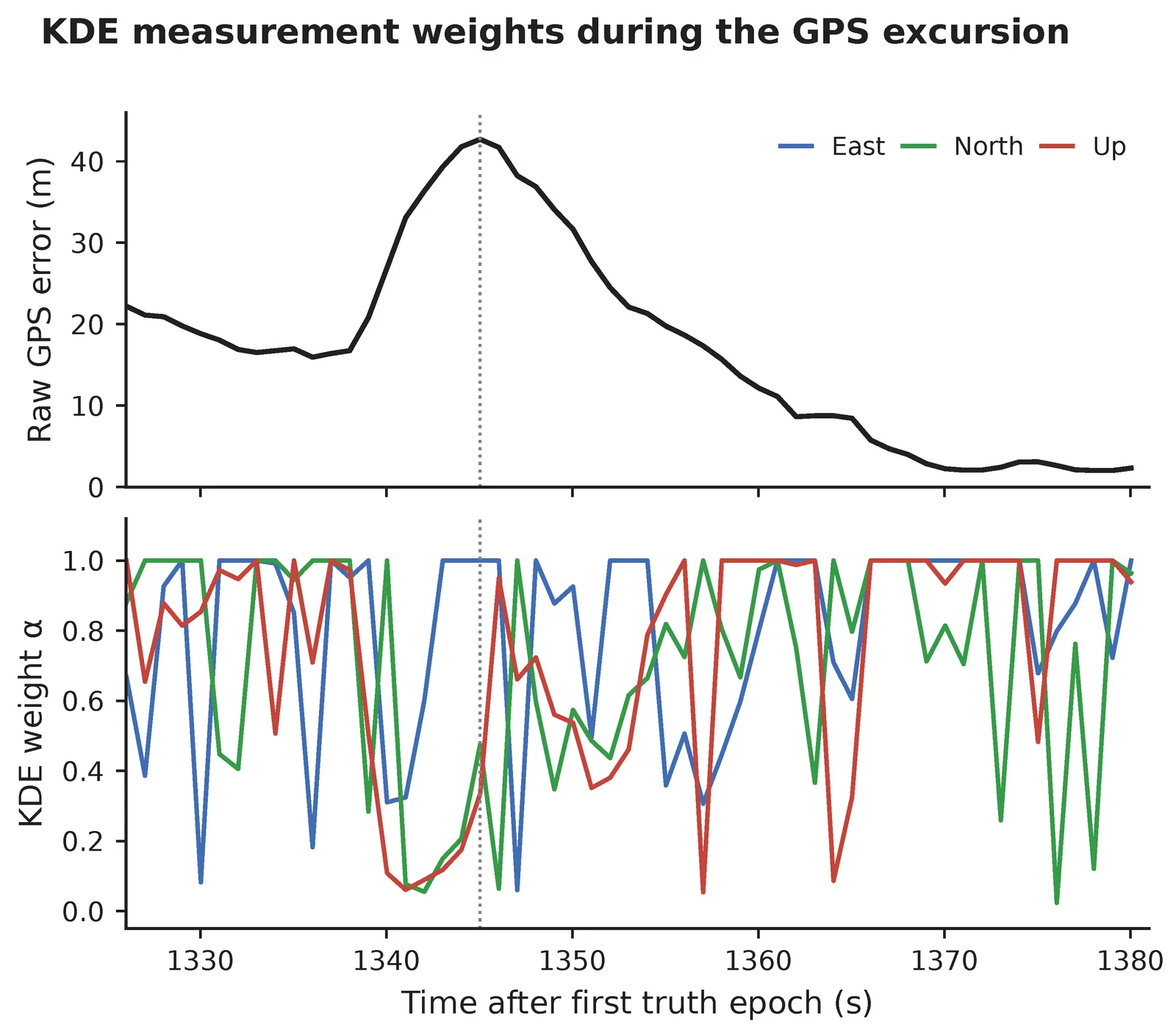
Channel-wise KDE measurement weights during the GPS excursion.

The adaptive mechanism underlying this behaviour is illustrated by the channel-wise KDE weights in Figure 9. Under nominal measurement conditions, the East, North, and Up weights remain close to unity, allowing GPS information to contribute normally to the measurement update. Around t=1345s, the weights decrease sharply in the affected channels as the corresponding GPS innovations move into low-density regions of their estimated distributions. Notably, the three weights do not decrease synchronously, indicating that the measurement degradation is direction-dependent rather than isotropic. As the innovations return to high-density regions, the weights recover towards unity and the GPS measurements progressively regain influence on the state update.

The temporal correspondence between the reduction in KDE weights and the suppression of the position-error excursion provides direct evidence of the intended robust-update mechanism. Rather than applying a fixed rejection threshold or a common parametric tail model, KDE-RKF continuously adjusts the contribution of each measurement channel according to the local density of its innovation. Consequently, anomalous GPS measurements are strongly down-weighted during degraded intervals, while statistically consistent measurements retain their contribution to the navigation solution.

## 4. Discussion

The simulations indicate that recent local innovation density can act as a measurement-reliability signal under the tested GNSS degradations. The increasing separation at higher contamination is consistent with the method’s use of local density rather than a single fixed tail model. The public urban MAV flight additionally demonstrates executable sensor-data integration without artificial contamination. KDE-RKF had the lowest held-out RMSE among the filtered solutions and reduced the peak 3D error by 12.66m relative to EKF during the largest natural excursion. Therefore, this single flight data only demonstrates a modest peak-error suppression capability.

For remote-sensing georeferencing, the soft-gating interpretation is valuable because it is continuous and channel-specific. GNSS degradation is rarely identical across horizontal position, vertical position, horizontal velocity, and vertical velocity. Multipath may primarily affect the vertical channel, while carrier tracking degradation may affect velocity differently from position. Channel-wise KDE weighting avoids rejecting an entire measurement vector when only part of it is unreliable.

The method also supports operational interpretability. The weight $\alpha_{k}$ can be logged as a measurement-quality indicator for downstream remote-sensing processing. Image frames, scanlines, or point-cloud segments acquired during low $\alpha_{k}$ intervals can be flagged for quality control or processed with larger geolocation uncertainty. Thus, KDE-RKF provides both navigation robustness and a transparent reliability signal.

Several limitations remain. First, the public field experiment contains one slow, tethered, low-altitude urban MAV flight rather than repeated highly dynamic fixed-wing or rotorcraft missions. Its approximately 1Hz photogrammetric truth, public synchronization, and unavailable mission-specific antenna and payload calibration do not permit separate identification of latency, lever-arm, or antenna effects. The 31 temporal blocks characterize within-flight variation and are not independent flight replicates, so a new trajectory is required for external confirmation. Second, the 150-epoch simulation startup period needs a dedicated error curve, and recovery after window reset has not been quantified. Third, channel-wise KDE does not model cross-channel dependence. Fourth, empirical NEES/NIS calibration and correlated-outlier tests are absent. Fifth, the fixed baseline settings were not optimized per scenario, and MCC-KF or another adaptive non-parametric baseline has not yet been included. Finally, the timing and memory analysis does not replace embedded or fixed-point validation.

## 5. Conclusions

This study developed KDE-RKF for GNSS/INS georeferencing under measurement outliers. The filter combines logarithmic Gaussian kernel density estimation, adaptive bandwidth selection, and channel-specific soft-gating to adjust the effective measurement covariance according to recent innovation statistics.

In Monte Carlo simulations, KDE-RKF reduced positioning errors under isolated outliers, attitude-related bursts, terminal multipath, and simulated jamming. At 15% isolated-outlier contamination, its three-dimensional position RMSE was 38.4m, compared with 87.3, 47.2, and 44.3m for EKF, Huber-KF, and VB-Student-KF, respectively. Positioning accuracy recovered to near-nominal levels within 1.8s after simulated jamming ended. The measured computation time was 0.312ms per GNSS update on the simulation platform. Ablation results showed lower RMSE following the successive inclusion of the logarithmic kernel, channel-specific weighting, and adaptive bandwidth selection.

Evaluation on the AGZ_subset sequence of the Zurich Urban Micro Aerial Vehicle dataset provided additional evidence under naturally degraded GPS measurements. During the reported GPS excursion, KDE-RKF reduced three-dimensional-position RMSE from 21.44 to 17.30m and peak error from 41.63 to 28.97m relative to EKF. Together, the simulation and urban-flight results support local innovation density as a basis for adaptive measurement weighting in GNSS/INS georeferencing. Further evaluation should examine repeated highly dynamic flights, cross-channel error dependence, and implementation on embedded hardware.

## Figures and Tables

**Table 1:** Simulation and Sensor Configuration.

Item	Value
Trajectory duration	115s highly dynamic flight
GNSS update rate	10Hz
IMU class	Tactical-grade MEMS, STIM300-class
Nominal GNSS horiz. accuracy	2.5m CEP
Nominal GNSS vert. accuracy	5m CEP
KDE window	N=150 GNSS epochs
Monte-Carlo runs	100 per scenario

**Table 2:** Summary of Benchmark Filters.

Method	Robustification Mechanism	Key Parameters
EKF	None	Nominal Q and R
$\chi^{2}$-EKF	Hard NIS threshold	0.99 chi-square threshold
Huber-KF	M-estimation	Huber constant c=1.345
VB-Student-KF	Heavy-tailed measurement model	v=4, 5 VB iterations
KDE-RKF	Non-parametric KDE soft-gating	N=150, log-Gaussian kernel

**Table 3:** Comparison of Scenario A 3D-Position RMSE (m). Values are presented as Mean ± Standard Deviation.

Contamination	EKF	Chi2-EKF	Huber-KF	VB-Student-KF	KDE-RKF
0%	18.4 ± 1.2	18.4 ± 1.2	18.6 ± 1.3	18.5 ± 1.2	18.5 ± 1.2
5%	38.7 ± 3.1	28.4 ± 2.2	25.1 ± 1.9	23.8 ± 1.7	22.4 ± 1.5
10%	62.5 ± 5.8	43.2 ± 3.6	37.3 ± 2.8	34.1 ± 2.4	30.5 ± 2.0
15%	87.3 ± 8.4	55.8 ± 5.1	47.2 ± 3.9	44.3 ± 3.3	38.4 ± 2.7
20%	121.6 ± 13.2	82.4 ± 8.3	68.7 ± 6.2	61.5 ± 5.1	48.1 ± 3.8

**Table 4:** Position-error statistics during the GPS excursion in AGZ_subset (1326.028–1380.033s).

Method	3D RMSE (m)	3D MAE (m)	95th-Percentile Error (m)	Peak 3D Error (m)	Peak Reduction vs. EKF (%)
Raw GPS	21.20	17.49	40.05	42.67	−2.50
EKF	21.44	17.95	39.97	41.63	0.00
$\chi^{2}$-EKF	18.99	15.81	36.61	40.52	2.66
Huber-KF	20.51	17.32	37.06	38.17	8.32
Student-*t* KF	20.14	17.19	35.31	36.28	12.86
KDE-RKF	17.30	14.81	28.45	28.97	30.42

## Data Availability

The data presented in this study are available on request from the corresponding author.

## Abbreviations

The following abbreviations are used in this manuscript:

GNSS	Global Navigation Satellite System
INS	Inertial Navigation System
IMU	Inertial measurement Unit
EKF	Extended Kalman Filter
MEMS	Microelectromechanical System
KDE	Kernel Density Estimator
RMSE	Root Mean Square Error
KDE-RKF	Kernel Density Estimate–Robust Kalman Filter
LiDAR	Light Detection and Ranging
